# No polymorphisms in K13-propeller gene associated with artemisinin resistance in *Plasmodium falciparum* isolated from Brazzaville, Republic of Congo

**DOI:** 10.1186/s12879-018-3453-6

**Published:** 2018-10-29

**Authors:** Pembe Issamou Mayengue, Roch Fabien Niama, Dezi Kouhounina Batsimba, Alida Malonga-Massanga, Igor Louzolo, Nadia Claricelle Loukabou Bongolo, Lucette Macosso, Reyna Ibara Ottia, Ghyslain Kimbassa Ngoma, Louis Régis Dossou-Yovo, Brice Pembet Singana, Gabriel Ahombo, Géril Sekangue Obili, Simon Charles Kobawila, Henri Joseph Parra

**Affiliations:** 1 Faculté des Sciences et Techniques, Université Marien Ngouabi, BP 69, Brazzaville, République du Congo; 2 Laboratoire National de Santé Publique, BP 120, Brazzaville, République du Congo; 3 Ecole Normale Supérieure, Université Marien Ngouabi, BP 69, Brazzaville, République du Congo; 4 Centre Hospitalier Universitaire de Brazzaville, BP 1846, Brazzaville, République du Congo

**Keywords:** *Plasmodium falciparum*, K13-propeller gene, Resistance, ACTs, Brazzaville, Republic of Congo

## Abstract

**Background:**

In the Republic of Congo, artemisinin-based combinations have been recommended for the treatment of uncomplicated malaria since 2006. However, the emergence of resistant parasites again these combinations in Southeast Asia is a threat for the control of this disease, especially in sub-Saharan Africa where the weight of the disease is important. Indeed, polymorphisms in *Plasmodium falciparum* K13-propeller gene have been involved in variations of drug sensitivity of *Plasmodium falciparum* to artemisinin-based combinations. The aim of the current study is to determine the prevalence of mutations of this gene in isolates collected in three health centers in Brazzaville.

**Methods:**

From May 2015 to May 2016, a total of 131, 259 and 416 samples from patients with suspected malaria were collected at the Laboratoire National de Santé Publique, Hôpital de Mfilou, and the CSI «Maman Mboualé» respectively. After DNA isolation, genotyping and sequencing of *Plasmodium falciparum* K13-propeller were performed in positive *Plasmodium falciparum* isolates identified after *msp-2* gene genotyping.

**Results:**

All 806 samples collected were *msp-2* genotyped and *Plasmodium falciparum* infections were confirmed in 287 samples with 43, 85, 159 samples from Laboratoire National de Santé Publique, Hôpital de Mfilou, and the CSI «Maman Mboualé» respectively. Of these 287 *msp-2* positives samples, K13-propeller nested PCR products were successfully obtained from 145 (50.52%) isolates and sequences were generated from 127(87.58%) nested products. None of mutations that were associated with ACTs resistance in Southeast Asia were detected on the samples from three different study sites from Brazzaville. However, one mutation type was observed at position 578, where alanine was substituted by serine (A578S) in two isolates (1.57%, 2/127), those from the Hôpital de Mfilou. No mutation was found in isolates from the two other sites.

**Conclusion:**

The current study shows a very limited polymorphism in the K13-propeller gene in isolates from the Republic of Congo and K13 polymorphisms associate with ACT resistance are not present in this country. However, permanent and large surveillance of resistant parasite population using K13-propeller gene is recommended.

## Background

In the Republic of Congo, malaria remains one of the main causes of consultation in health centers, with children under 15 years old and pregnant women being the most vulnerable groups [1, 2]. Like in almost all sub-Saharan African countries, the high level of resistance of *Plasmodium falciparum* to chloroquine as well as the inefficacy of sulphadoxine-pyrimethamine and amodiaquine either singly or in combination for the treatment of uncomplicated malaria have led the Republic of Congo to change its anti-malarial drug policy for treating uncomplicated malaria to artemisinin-combination therapies (ACTs) in 2006 [3]. Previous studies have shown that ACTs remain highly efficacious in Sub-Saharan Africa, including the Republic of Congo [4–7]. However, the emergence of resistance to artemisinin derivatives, including ACTs in Southeast Asia is a serious public health concern. If resistance to ACTs may spread, particularly in sub-Saharan Africa, the public health implications could be disastrous, because no alternative drug is currently available with the same level of efficacy and tolerance than ACTs. In order to limit the development of *Plasmodium falciparum* resistance to current antimalarial, the World Health Organization (WHO) has recommended a vigilant surveillance of resistant parasites [8]. Recently, five principal mutations, namely the M476I, Y493H, I543T, R539T and C580Y in the *Plasmodium falciparum* Kelch propeller domain (K13-propeller) have been found to be associated with delayed parasite clearance after ACTs therapy in Cambodia [9]. Although no mutations associated with prolonged artemisinin clearance in Southeast Asia are found in sub-Saharan African regions, while diverse others mutations are identified even in isolates collected before or after the introduction of ACTs, regular surveillance is needed, taking into consideration the critical importance of ACTs in the control and elimination of malaria in these regions [10–14]. Thus, the current study aimed at measuring the prevalence of *Plasmodium falciparum* K13-propeller polymorphisms in clinical isolates collected from Brazzaville 10 years after the introduction of ACTs.

## Methods

### Study areas and site preparation

Brazzaville, the political capital hosts 38% (1,642,105 inhabitants) of the total population of the Republic of Congo**,** estimated at 4312715 inhabitants. With the population expansion due to urbanization, Brazzaville is now divided into nine districts: Bacongo, Makelekele, Poto-Poto, Moungali, Ouenze, Talangaï, Mfilou, Madibou and Djiri. The present study was conducted in three health centers: Centre de Santé Intégré (CSI) « Maman Mboualé» located in the district of Talangaï, in the north part of city (4°13’S, 15°17′E); Hôpital de Mfilou located in the district of Mfilou, in the south part of the city (4°15’S, 15°13′E) and the Laboratoire National de Santé Publique (LNSP), the national reference laboratory located in the center part of city, in the district of Poto-Poto (4°16’S, 15°15′E).

Malaria transmission in the study areas varies from low, moderate to intense with meso-, hyper- to perennial endemicity. Malaria infection is primarily due to *P. falciparum*. Two rainy seasons are observed each year with the main one during the months of February to May, and a short one from October to December [15].

In the early project stage, first, a meeting with the site actors including the head of each laboratory and microscopists was organized for the purpose of presenting the project objectives, methodology and expected results, as well as to obtain microscopists consent.

### Study population, blood samples and data collection

From May 2015 to May 2016, patients with clinical signs of uncomplicated malaria, presenting at the laboratory of one of the three study sites were invited to participate in this study. Exclusion criteria comprised pregnancy, severe malaria or other severe illness as judged by the attending physician. A number of representative patients to be included each month, per week and per day has been estimated by the statistician taking into account the proportion of malaria reported in each health center, 1 year before starting the study. In sample size calculations, considering the proportion of malaria at 73.29% for the CSI « Maman Mboualé», 87.75% for Hôpital de Mfilou and 2.33% for LNSP, using a confidence level of 95% and a marge error of 5%, the SCHWARZ method [16] yielded a minimum number of 310, 200 and 100 patients to be recruited at the CSI « Maman Mboualé», Hôpital de Mfilou and the LNSP, respectively. Recruited patients were randomly selected from Monday to Friday. At the CSI « Maman Mboualé», a minimum of 7 patients were recruited per week, with at least one patient per day, whereas, at the Hôpital de Mfilou, the minimum number was 5 patients per week, with one per day. For the LNSP, 3 patients were enough, with one included on Monday, on Wednesday and on Friday. After informed consent was obtained, records were made on patient demographics, fever or history of fever in the last 48 h, other signs of malaria, provenance, previous antimalarial drugs intake used of bed net treated. The axillary temperature was taken for fever confirmation.

At each study site, blood sample from each patient was blotted on the Whatman filter paper (3MM CHR) while preparing the thick blood smears, dried and transferred to the “Laboratoire National de Santé Publique” in Brazzaville, where isolation of deoxyribonucleic acid (DNA), polymerase chain reaction (PCR) and sequencing were performed. Before reading, thick blood smears were dried and stained with 10% Giemsa solution (Sigma Chemical, Sigma Aldrich ChemieGmbh, Taufkirchen, Germany) in pH 7.2, for approximately 10 min. The stain was gently washed away by adding drops of clean water and the slide was completely dried before examination. Thick blood smears were assessed by micrsocopists until 200 leucocytes had been counted. Parasite density was calculated for each patient assuming an average of 8000 leucocytes per μl of blood using the proposed method of the WHO [17]. Individual diagnostic result was given to each patient and advised to meet the prescribers for possible antimalarial chemotherapy.

### Extraction of parasite DNA

Genomic DNA was extracted from samples collected on the Whatman filter paper by QIAamp DNA mini Kit (Qiagen, Hilden, Germany) according to the manufacturer’s instruction. Briefly, 3 circles of approximately 3 mm diameter were punched out from a blood spot and placed into a 1.5 mL micro centrifuge tube in which 180 *μ*L of ATL buffer was added. Successive washing steps with AW1 buffer and AW2 buffer were followed by DNA elution with 150 *μ*L of AE buffer. Extracted DNA was stored at − 20 °C until use.

### Genotyping

For multiple purposes, the highly polymorphic central region of *Merozoite Surface Protein-2* (*msp-2*) gene was firstly genotyped as described previously [18], and positive *Plasmodium falciparum* samples were used for K13- propeller genotyping. PCR amplification was performed following a 2-step amplification procedure in which the initial amplifications were followed by the nested PCR reactions using specific primers K13–1 and K13–4 for the first round and K13-inF2 and K13-inR2 for the nested PCR (Table 1), flanking the codons T474I, M476I, A481V, Y493H, T508 N, P527T, G533S, N537I, R539T, I543T, P553L, R561H, V568G, P574L, A578S, and C580Y; as described by Li et al. [12]. The amplification reactions were carried out as described by Ariey et al. [9]. For the first round, 1 μL of DNA was amplified with 200 μM of each deoxynucleotide triphosphate (dNTP), 1 μM of each primer, 3 mM MgCl_2_, 1.5 units of Taq DNA polymerase and sterile ultrapure water to a final reaction of 25 μL. The mixtures were denatured at 95 °C for 5 min, followed by 40 amplification cycles (94 °C for 30 min, 60 °C for 1 min 30 s, and 72 °C for 1 min 30 s), and final elongation at 72 °C for 10 min. For the second round PCR, similar procedure with specific primers was used and 5 μL of the first PCR product were used as template.

**Table 1:** K13- propeller primers sequences

Primer name	Sequences 5′……………………………………………3′	Size (bp)
K13–1(forward)	CGGAGTGACCAAATCTGGGA	2097
K13–4 (reverse)	GGGAATCTGGTGGTAACAGC	
K13-inF2 (forward)	TCAACAATGCTGGCGTATGTG	501
K13-inR2 (reverse)	TGATTAAG GTAATTAAAAGCTGCTCC	

All PCR products were analyzed using 2% agar gel electrophoresis. Thereafter PCR products were purified using PureLink™ Quick PCR Purification Kit (Invitrogen, by Thermo Fischer Scientific) and directly used as templates for DNA sequencing using an ABI 3500xL automated sequencer (Applied Biosystems Genetic Analyzer, HITACHI). The data was analyzed using Geneious 10.2.3 [19]. Mutations were assessed by comparing each sequence with the 3D7 K13-propeller (PF3D7_1343700) used as the reference.

### Data analysis

Data were entered and verified in Microsoft Excel (Microsoft Corp., Seattle, USA) and validated in EpiInfo for Windows version 3.5.1. Data were analyzed using the SPSS 16.0 for Windows (Inc., Chicago, USA).The mutant and wild-type alleles identified in the analyzed isolates were used to generate the prevalence of the alleles.

## Results

### Characteristics of febrile patients with symptoms of malaria

A total of 131, 259 and 416 patients with suspected malaria were enrolled at the LNSP, Hôpital de Mfilou and the CSI «Maman Mboualé» respectively.

Out of 259 patients enrolled at the Hôpital de Mfilou, gender and age were recorded for 257 of them, while with regards to the CSI « Maman Mboualé», out of the 416 recruited patients, 410 had records on gender (Table 2).

**Table 2:** Characteristics of patients

Characteristics	LNSP	Hôpital de Mfilou	CSI « Maman Mboualé»
Total numbers	131	259	416
Gender (F/M)	68/63	131/126	207/203
Median age (years)	42 (11–74)	26 (0.5–84)	10 (0.5–75)
Groups of age (%)			
< 5 years	0 (0.0)	38 (14.8)	99 (23.8)
≥ 5 years	131 (100)	219 (85.2)	317 (76.2)

All 806 samples collected were *msp-2* genotyped and *Plasmodium falciparum* infections were confirmed in 287 samples with 43, 85, 159 samples from LNSP, Hôpital de Mfilou, and CSI «Maman Mboualé» respectively. These *msp-2* positive samples were then considered for K13-propeller genotyping and nested PCR products was successfully obtained from 145 (50.52%) isolates, with 5, 50 and 90 from LNSP, Hôpital de Mfilou, and CSI «Maman Mboualé» respectively (Table 3). K13-propeller sequences were generated from 127(87.58%) nested products (17 isolates did not generate good quality sequences that could be analyzed). To analyze polymorphisms in the K13-propeller, sequences alignment was done by comparing with the 3D7 strain (PF3D7_1343700).

**Table 3:** Identification of *Plasmodium falciparum* isolates and total number of K13-propeller sequence

Sites	Number of *Plasmodium falciparum* isolates	Number of K13 nested PCR	Number of K13-propeller sequence
LNSP	43	5 (11.62%)	3 (60%)
Hôpital de Mfilou	85	50 (58.82%)	48 (96%)
CSI « Maman Mboualé »	159	90 (56.60%)	76 (84.4%)
Total	287	145 (50.52%)	127 (87.58%)

None of mutations that were associated with ACTs resistance in Southeast Asia were detected on the samples from three different study sites from Brazzaville. However, one mutation type was observed at position 578, where alanine was substituted by serine (A578S). Only two isolates (with the frequency of 1.57%, 2/127) had carried the A578S substitution, and those from the Hôpital de Mfilou. No mutation was found in isolates from CSI « Maman Mboualé » and LNSP.

## Discussion

The present study of which all steps were carried out in Brazzaville, including K13-propeller sequencing, allowed us to analyze the polymorphism related to ACTs resistance in 127 *Plasmodium falciparum* isolates from Brazzaville. In the Republic of Congo, ACTs are recommended as first- and second-line treatment for uncomplicated malaria since 2006, and the efficacy of these combinations remains high as reported by Ndounga et al. [5, 6]. However, it is important to monitor the eventual occurrence of the ACTs-resistant parasite population. The causes of the occurrence of resistance are multiple, including self-medication, which does increase drug pressure. Although the WHO recommendations require a preliminary parasitological test before taking any ACT, it is not surprising to notice the intake of these drugs without medical prescription in Brazzaville. Resistance to artemisinin and its derivatives is considered as major risk to public health, which would have a greater impact in sub-Saharan Africa where the burden remains high. The emergence of *Plasmodium falciparum* resistance to artemisinin and its derivatives, which appear as delayed parasite clearance after ACT treatment, has been reported in Southeast Asia [20]. At the molecular level, five major mutations in the *Plasmodium falciparum* K13-propeller gene, namely the M476I, Y493H, I543T, R539T, and C580Y mutations have been found to be associated with in vitro resistance to artemisinin and delayed parasite clearance following treatment with ACTs [9]. However, in the current study none of these mutations were found. Moreover, only a single limited A578S mutation has been found only in two isolates, all from Hôpital de Mfilou. A very low proportion of this A578S mutation has been also reported in Cambodia by Straimer et al. [21] and in many sub-Saharan countries such as Uganda, Mali, Equatorial Guinea, Kenya, Democratic Republic of Congo, and Ghana [10–12, 22–24].

The treatment outcomes and parasite clearance profiles for patients with A578S mutations in the current study were not evaluated. However, study conducted in Mali has shown that parasite clearance time was comparable between infections with non-synonymous K13-propeller mutations (including A578S mutation) and infections with the reference allele [11]. Thus, due to the common presence of this mutation in many countries, further characterization is needed as well as assessment of the role of this mutation in in vivo parasite clearance in others sub-Saharan Africa countries including the Republic of Congo is required.

Furthermore, the A557S mutation detected in the Republic of Congo by Taylor et al. [25] has not been detected in the current study. None of the 13 new mutations not associated with resistance to ACTs identified in the Republic of Congo by Koukouikila-Kousounda et al. [14] were also identified in the current study. Taking into account this large difference, repeated studies including different sites in all departments and increasing the number of isolates to be analyzed, are needed to better characterize the actual profile of polymorphisms in the K13- propeller gene in this country.

Although none of the mutations associated with artemisinin resistance in South-East Asia are present in sub-Saharan Africa including the Republic of Congo, new mutations are emerging on the African continent [26]. Thus, the impact of these mutations on resistance to ACTs is to be explored.

## Conclusion

The current study shows very limited polymorphism in the K13-propeller gene in clinical isolates from the Republic of Congo and polymorphisms associate with ACTs resistance are not present in this country. Only one mutation type was observed at position 578, where alanine was substituted by serine (A578S) in two isolates, those from the Hôpital de Mfilou. However, permanent and large surveillance of resistant parasite population using K13-propeller gene is recommended.

## Abbreviations

ACTs: artemisinin-combination therapies
CERSSA: Comité d’Ethique de la Recherche en Sciences de la Santé
CSI: Centre de Santé Intégré
DNA: deoxyribonucleic acid
dNTP: deoxynucleotide triphosphate
LNSP: Laboratoire National de Santé Publique
PCR: polymerase chain reaction
WHO: World Health Organization
